# A general urban spreading pattern of COVID-19 and its underlying mechanism

**DOI:** 10.1038/s42949-023-00082-4

**Published:** 2023-01-28

**Authors:** Hongshen Zhang, Yongtao Zhang, Shibo He, Yi Fang, Yanggang Cheng, Zhiguo Shi, Cunqi Shao, Chao Li, Songmin Ying, Zhenyu Gong, Yu Liu, Lin Dong, Youxian Sun, Jianmin Jia, H. Eugene Stanley, Jiming Chen

**Affiliations:** 1 College of Control Science and Engineering, Zhejiang University, Hangzhou, China; 2 Westlake Institute for Data Intelligence, Hangzhou, China; 3 College of Information Science and Electronic Engineering, Zhejiang University, Hangzhou, China; 4 Key Laboratory of Collaborative sensing and autonomous unmanned systems of Zhejiang Province, Hangzhou, China; 5 School of Medicine, Zhejiang University, Hangzhou, China; 6 Zhejiang Provincial Center for Disease Control and Prevention, Hangzhou, China; 7 Shenzhen Finance Institute, School of Management and Economics, The Chinese University of Hong Kong, Shenzhen, China; 8 Center for Polymer Studies and Physics Department, Boston University, Boston, MA 02215 USA

**Keywords:** Social sciences, Diseases

## Abstract

Currently, the global situation of COVID-19 is aggravating, pressingly calling for efficient control and prevention measures. Understanding the spreading pattern of COVID-19 has been widely recognized as a vital step for implementing non-pharmaceutical measures. Previous studies explained the differences in contagion rates due to the urban socio-political measures, while fine-grained geographic urban spreading pattern still remains an open issue. Here, we fill this gap by leveraging the trajectory data of 197,808 smartphone users (including 17,808 anonymous confirmed cases) in nine cities in China. We find a general spreading pattern in all cities: the spatial distribution of confirmed cases follows a power-law-like model and the spreading centroid human mobility is time-invariant. Moreover, we reveal that long average traveling distance results in a high growth rate of spreading radius and wide spatial diffusion of confirmed cases in the fine-grained geographic model. With such insight, we adopt the Kendall model to simulate the urban spreading of COVID-19 which can well fit the real spreading process. Our results unveil the underlying mechanism behind the spatial-temporal urban evolution of COVID-19, and can be used to evaluate the performance of mobility restriction policies implemented by many governments and to estimate the evolving spreading situation of COVID-19.

## Introduction

As of December 12, COVID-19 has struck over 222 countries, resulting in >630 million confirmed cases and >6.5 million deaths^1^. The maximum of global daily new cases have exceeded 1,000,000, and situations in many countries are increasingly aggravating. It is pressing to find efficient ways to suppress the transmission of SARS-CoV-2^2–11^, which has been recognized as top priority since the very beginning of the initial outbreak in China and reiterated by the multidisciplinary online conference on 3 August 2020, organized by the World Health Organization^12^.

Recently, resurgences of COVID-19 have been reported in many countries (e.g., United Kingdom, France, Spain). When a local resurgence takes place^13–15^, a fundamental issue for practical control and prevention is how does COVID-19 spread temporally and spatially within a city? Many works have explained the differences in contagion rates due to the urban socio-political measures. Manzira et al. presents a strong relationship between modes of transportation(such as traffic volume, bus passengers, pedestrians, and cyclists) and reported COVID-19 infections^16^. Lak et al. suggested that the demographic composition and major neighborhood-level physical attributes are important factors explaining high infection rates and mortality^17^. Ma et al. revealed the policy of mask use in controlling the transmission of COVID-19^18^.

Existing works focusing on the spreading pattern of COVID-19 can be summarized into two categories based on the spatial scale: the spreading of COVID-19 within a specific city or between different cities or countries. Considering spreading within a specific city, Hamidi found that the metropolitan population is one of the most significant predictors of infection rates^19^. Acuto et al. proposed that urban equality can engender healthier and more sustainable societies^20^. Sharifi et al. introduced the impacts of COVID-19 on environmental quality, socio-economic impacts, management and governance, and transportation and urban design^21^. Gaisie argued that the evolution of the COVID-19 through built environment attributes such as diversity, destination accessibility, distance to transit, design, and density^22^. For spreading between cities, the relatively wide COVID-19 spreading pattern focuses on the inter-country, inter-state, or inter-districts spreading pattern of diseases. Due to the lack of fine-grained data, previous works^23–25^ are always not able to compare with real data of confirmed cases and medical virus detection can not track the daily trajectory of ten thousands or more confirmed cases. They fall short on studying fine-grained urban transmission dynamics of COVID-19, one of the most critical spreading characteristics.

Here, we fill this gap by leveraging the trajectory data of 197,808 smartphone users (including 17,808 anonymous confirmed cases) in 9 cities in China. To have a comprehensive analysis, we select confirmed cases from Wuhan (where the initial outbreak of COVID-19 took place in China), Beijing and Urumqi (the cities with COVID-19 resurgences) and other cities (where cases were mainly imported). We explain the spreading process through the following three aspects: (1) The general spreading pattern of COVID-19 in different cities; (2) The underlying mechanism for the spreading pattern of COVID-19; (3) The utilization of the spreading pattern for control and prevention of COVID-19. We find a general spreading pattern existing in all cities: the spatial distribution of confirmed cases follows a truncated power-law-like model and the spreading centroid is time-invariant. Moreover, we reveal that long average traveling distance results in a high growth rate of spreading radius and wide spatial diffusion of confirmed cases in the fine-grained geographic model. With such insight, we can accurately predict the shapes of spatial distribution of cases and the time when the peak of COVID-19 cases arrives. Our results unveil the underlying mechanism behind the spatial-temporal urban evolution of COVID-19, and can be used to evaluate the performance of mobility restriction policies implemented by many governments and to estimate the evolving spreading situation of COVID-19.

## Results

### The temporal spreading pattern

**Fig. 1:**
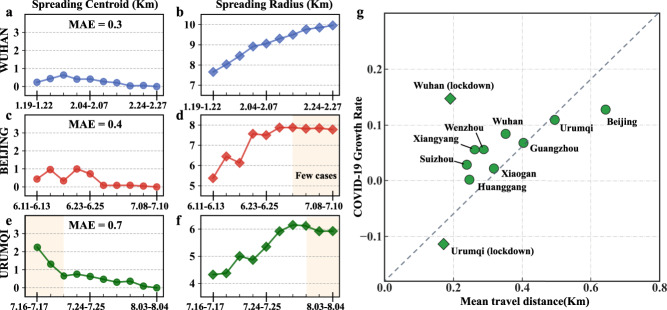
The temporal spreading pattern of COVID-19. **a**–**f** The cumulative spreading centroid and spreading radius in Wuhan, Beijing, and Urumqi, respectively. **g** Relation between the mean travel distance of people in each city and the corresponding COVID-19 growth rate.

We display the difference between the overall spreading centroid and cumulative spreading centroid until *i*th period in Fig. 1a, c, and e for Wuhan, Beijing, and Urumqi, respectively. Interestingly, as the situation of COVID-19 evolves, cumulative spreading centroids at different periods in Wuhan, Beijing, and Urumqi are close to the overall spreading centroid: the mean absolute errors (MAE) between cumulative and overall spreading centroids in Wuhan, Beijing, and Urumqi are 0.3 Km, 0.4 Km, and 0.7 Km, respectively. It is clear to see that the temporal spreading centroid of COVID-19 has a feature of time invariance. That is, the spreading centroid is stable and nearly does not migrate during COVID-19 spreading.

We also conduct analysis for the other six cities with a relatively large number of confirmed cases in China: Xiaogan, Suizhou, Xiangyang, Huanggang, Guangzhou, and Wenzhou (Supplementary Fig. 6), where cases are mainly imported. Similar conclusions for spreading centroid and spreading radius can be made in these cities. We proceed to perform sensitivity analysis by varying the number of spreading period *L* (Supplementary Fig. 5) and find that *L* does not have much impact on the observed temporal pattern. Therefore, the temporal spreading pattern of COVID-19 in China features time invariance of spreading centroid and slow growth of spreading radius.

**Fig. 2:**
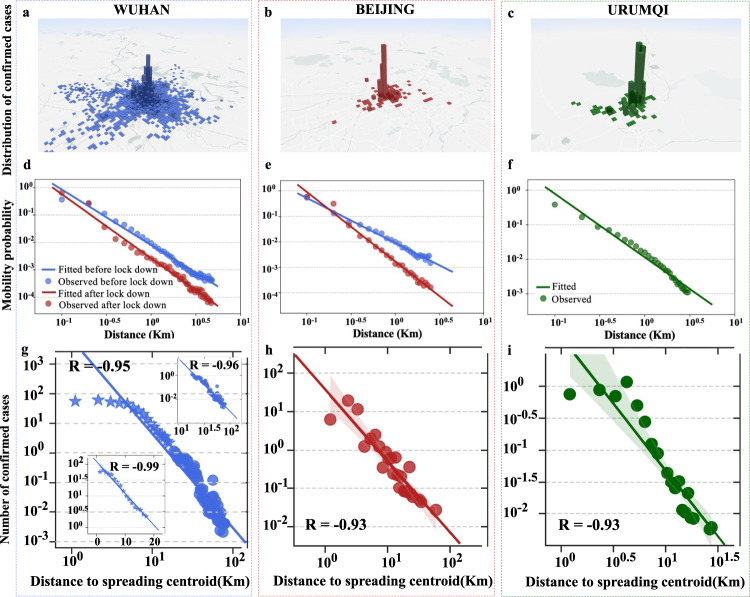
The spatial spreading pattern of COVID-19 in Wuhan, Beijing, and Urumqi. **a**–**c** A visualization of the number of confirmed cases in discretized grids in Wuhan, Beijing, and Urumqi, respectively. **d**–**f** The human mobility distribution (dots) as a function of traveling distance and the fitted models (lines) for Wuhan, Beijing, and Urumqi, respectively. **g**–**i** The spatial distributions (dots) as a function of distance from the overall spreading centroid and the fitted regression lines for these distributions.

As can be seen in Fig. 1 and Supplementary Fig. 4, there are significant disparities in growth rate of spreading radius in different cities and different time periods. To find intrinsic mechanisms for these disparities, we first divide the spreading period *T* (*T* in Beijing for instance lasted from June 11 to July 10 with 30 days) of each city into two periods *L*_1_ and *L*_2_ (*L*_1_ in Beijing for instance lasted from June 11 to June 25 with 15 days). Then, two spreading radii (*R*_1_ and *R*_2_) can be calculated based on activity centroids of confirmed cases reported in the spreading periods *L*_1_ and *L*_2_, respectively. We define the growth rate of spreading radius as 2(*R*_2_−*R*_1_)/∣*T*∣, where ∣*T*∣ denotes number of days in spreading period *T*. Further, we randomly select 20,000 smartphone users in each city and leverage their trajectory data during the outbreak of COVID-19 to compute their mean travel distance. Since these smartphone users are randomly selected, we use this mean distance (over 20,000 users) to approximate that of all citizens in each city. Clearly, a large value of mean travel distance reflects a strong willingness of people for long-distance travelling. Considering that different control measures imposed in Wuhan and Urumqi since the outbreak of COVID-19 affected the corresponding mobility pattern and spreading of pandemic significantly (Fig. 2d, e), we divide the spreading period of these two cities into two sub-periods: before and after the implementation of travel restriction, and then calculate mean travel distance and growth rate of spreading radius in these two sub-periods, respectively. The correlation analysis results for all nine cities are illustrated in Fig. 1g. Interestingly, we observe a clear positive correlation between mean travel distance and growth rate of spreading radius, indicating that mobility pattern accelerates the urban spreading of COVID-19.

### The spatial spreading pattern

To characterize and visualize spatial spreading pattern, we divide the geographical area into grids of 1 Km × 1 Km^26^. The overall spreading centroid is set as the original point of grids. Confirmed cases of COVID-19 are then projected into grids according to their activity centroids. As illustrated in Fig. 2a–c, three-dimensional histograms are used to describe the spatial distributions of confirmed cases in Wuhan, Beijing, and Urumqi, respectively, where the height of each bar represents the case count in each grid.

To analyze the spatial distribution function *F*(*d*), driven by human mobility pattern, we apply the logarithm to the actual distribution of human mobility pattern as well as the number of confirmed cases and the distance from the overall spreading centroid in all cities. The human mobility distributions and spatial distributions *F*(*d*) of Beijing, Urumqi, Xiaogan, Suizhou, and Huanggang exhibit a prominent linear pattern (Fig. 2d–i, Supplementary Figs. 6 and 7). Although a significant change in human mobility patterns in Wuhan (Jan. 23) and Urumqi (Jul. 16) after control measures can be observed intuitively, corresponding human mobility distributions and spatial distributions in these cities are surprisingly demonstrated as power-law model. Therefore, we adopt *F*(*d*) = *d*^*α*^ for linear regression. Specifically, we have *α* = −1.80 for Beijing (the Pearson correlation: −0.93) and *α* = −2.15 for Urumqi (the Pearson correlation: −0.93), respectively.

As shown in Fig. 2g, the spatial distribution of confirmed cases in Wuhan is power-law-like since it deviates slightly from power-law model when *d* is small. Due to the influence of human mobility patterns in Wuhan during the lockdown period (Fig. 2d), initial cases have a higher probability to infect susceptible individuals around the spreading centroid. As a result, the risk of infection around spreading centroid is much higher than that at distant locations (a significant 92% of cases are close to the spreading centroid). To have a more accurate quantification of the spatial distribution of Wuhan, we divide the area into two parts by their distance to the spreading centroid, and adopt two different models to fit the data. Specifically, spatial distribution of confirmed cases around the spreading centroid (*d* ≤ 18) is fitted by an exponential model *F*(*d*) = *α*^*d*^, and when *d* > 18 is fitted by a power-law model. As illustrated in Fig. 2g, *F*(*d*), *d* ≤ 18 is well fitted by an exponential model with a Pearson correlation of −0.99, and *F*(*d*), *d* > 18 is well fitted by a power-law model with a Pearson correlation of −0.96. This indicates that the spatial distribution of confirmed cases in Wuhan indeed has different characteristics, depending on the distance to the spreading centroid. We also observe a similar phenomenon in Xiangyang (Supplementary Fig. 7f). Interestingly, we find that this is mainly determined by human mobility pattern (to be elaborated on in the next Section).

We also notice that for cities (such as Guangzhou and Wenzhou) where imported cases are widely scattered, the spatial spreading pattern is less prominent. It is clear that there are multiple clusters of confirmed cases in these two cities (Supplementary Fig. 8), which impacts the power-law-like spatial spreading. Therefore, the observed spreading pattern does not apply to the case with multiple infection sources.

### The underlying mechanism

The classic susceptible-infected-recovered model (SIR) and its variants have been widely adopted to understand the transmission characteristics of infectious diseases. The Kendall model^27–29^ introduces the spatial dimension to the SIR model and can be used to explain the spatial-temporal evolution of infectious diseases. Its differential equations can be expressed as equations (1)–(3) of supplementary materials. Note that confirmed cases in China get isolated for medical treatment once they are confirmed and would not cause further infection. Under such a condition, the confirmed cases can be regarded as recovered individuals in the Kendall model. Then, the differential equation for the proportion of recovered individuals can be written as

1 $$\frac{\partial R}{\partial t}=-\lambda R(x,t)+\lambda {I}_{0}(x)+\lambda \left[1-\exp \left(-\frac{1}{\lambda }\int\nolimits_{-\infty }^{\infty }R(y,t)K(x-y){{{\rm{d}}}}y\right)\right].$$

where *R*(*x*, *t*) denotes the proportion of recovered individuals at location *x* and time *t*, satisfying *R*(*x*, 0) = 0. Note that *λ* can be obtained by inverse of the basic regeneration number *R*_0_ in the model, that is, *λ* = 1/*R*_0_ = *γ*/*βξ*, where *γ*, *β*, *ξ* represents recovery rate, infection rate and the number of initial susceptible individuals, respectively. Besides, the kernel function *K*(*x* − *y*) > 0, satisfying $\int\nolimits_{-\infty }^{\infty }K(y){{{\rm{d}}}}y=1$, quantifies the probability that an infected individual at location *y* visits *x*. Here, we use power-law distribution to describe the city-level movement behaviors^30^, which can be written as *K*(Δ*r*) = Δ*r*^*η*^. Hereby, *K*(Δ*r*) represents the probability for the step size Δ*r* and *η*, the power-law exponential, denotes the travel willingness, which has a strong correlation with the mean traveling distance.

To fit the model for recovered individuals, we first calculate the parameter *η* in the power-law distribution by utilizing the mobility data of anonymous smartphone users, with which to capture the inherent human movement behaviors for each city. Moreover, the diagnosed date for each confirmed case and corresponding activity centroid is also calculated as input of the model. Through fitting the model (i.e., equation (1)) based on these precalculated parameters and Least Squares algorithm, we can finally obtain a set of optimal parameters (*λ* and *I*_0_(*x*)). Note that we assume initial confirmed cases originate from the grid (0, 0). The parameter *I*_0_(*x*) fitted in the model could therefore be written as *I*_0_(0, 0). A detailed discussion about Kendall model and parameter fitting process are provided in supplementary materials.

**Fig. 3:**
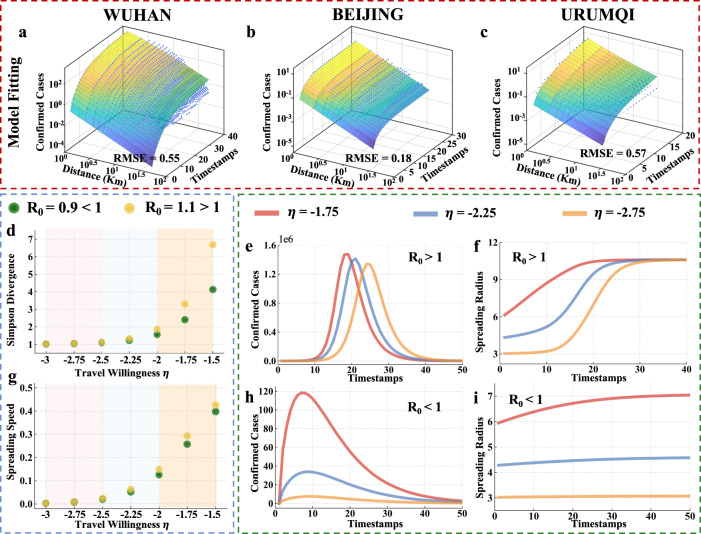
The spatial-temporal model for COVID-19 spreading pattern. **a**–**c** The overall model fitting performance. **d** Relation between travel willingness *η* and Simpson divergence. **e**, **h** Relation between travel willingness *η* and daily reported confirmed cases. **g** Relation between travel willingness *η* and the growth rate of spreading radius. **f**, **i** Relation between travel willingness *η* and spreading radius.

Figure 3a–c illustrate the overall model fitting performance of recovered individuals *R*(*x*, *t*) for Wuhan, Beijing, and Urumqi, in which Root-Mean-Square-Error (RMSE) is also added to quantify the performance of model fitting. The values of RMSE for Wuhan, Beijing, and Urumqi during whole spreading period are 0.55, 0.18 and 0.57 respectively, indicating that the evolution of recovered individuals *R*(*x*, *t*) during spreading period can be well captured by the proposed Kendall model. More results on the performance of model fitting can be found in Supplementary Fig. 9.

We proceed to study the impact of parameter *η* in the model on spatial dispersion of confirmed cases, the number of daily new confirmed cases, and growth rate of spreading radius during the whole spreading period. To characterize the spatial dispersion of confirmed cases, we introduce the concept of Simpson Divergence: $div=1/{{{\Sigma }}}_{i}{p}_{i}^{2}$, where *p*_*i*_ represents the proportion of confirmed cases distributed in grids whose distance to overall spreading centroid is within [*i*, *i* + 1]. Therefore, a small value of *div* reflects a high clustering of the confirmed cases, i.e., a large proportion of confirmed cases are distributed in a small number of grids. Fig. 3d illustrates the impact of *η* on spatial dispersion of confirmed cases. We consider two scenarios under different basic regeneration number *R*_0_: *R*_0_ < 1 and *R*_0_ > 1. We see that, with the decrease of *η* Simpson Divergence decreases to 1, at which all confirmed cases are distributed in one grid. The impact of *η* on growth rate of spreading radius is illustrated in Fig. 3g. Clearly, the growth rate of spreading radius decreases with the decrease of *η*, which is consistent with results in Fig. 3d. Figure 3e and h show the impact of *η* on the number of daily reported confirmed cases. Interestingly, a large *η*, which means long mean travelling distance in the human mobility model, results in quick spreading of COVID-19, which makes the peak of daily reported cases arrives early. This also shows that travel restriction policies will delay the peak arrival. Finally, we study the impact of *η* on the growth rate of spreading radius. As we can see in Fig. 3f, i, spreading radii under different *η* increase with time and converge to a fix value when *R*_0_ > 1. However, when *R*_0_ < 1, the spreading radius increases only when *η* is relatively large. This indicates that when *R*_0_ < 1 and the mean travelling distance is also low, the pandemic will not spread spatially. Therefore, *η* in the mobility model drives the temporal-spatial spreading process. In practice, we can optimize *η* by implementing a specific travel restriction policy to have a desired control and prevention performance.

## Discussion

Previous studies investigated the relationship between human mobility and spreading patterns of infectious diseases^31–33^, revealing that the number of infected cases at the destination has a strong correlation with the total population and the weighted distance from the source to the destination, which is determined by the corresponding population flow. Combining the population flow data and epidemic simulation model, these works accurately characterize large-scale spatial-temporal spreading of epidemics^34^ and predict future spreading trends^35–37^. Since the population flow is driven by human mobility, existing works have examined the intrinsic mechanism of how human mobility impacts the spreading of diseases^38–40^ and provided theoretical insights about how to mitigate transmission of epidemics through travel restriction^41–43^.

Interestingly, many urban patterns such as urban growth patterns, human mobility patterns, and income patterns^39,44–47^ follow the power-law distribution model. Since the a strong connection between the human mobility pattern and the spreading pattern, we would intrinsically guest that the spreading pattern has a similar distribution to the human mobility pattern and our result can also be viewed as complementary to the power-law distribution for urban pandemic spreading patterns.

Due to the lack of fine-grained data, previous works are always not able to compare with real data of confirmed cases and medical virus detection can not track the daily trajectory of ten thousand or more confirmed cases. Most works need ideal assumptions to reconstruct the whole fine-grained spreading process. In this article, we assume that individuals in China are homogeneous in the downtown area(less than 100 kilometers) and highly compliance with government regulations during the most severe spreading time(less than one month) with a constant infection rate and the same human mobility patterns before and after the lockdown policy. We use the data of confirmed cases’ activity centroids to study the spatial-temporal spreading pattern of COVID-19 in China. The novelty of our model is based on the trajectory data of anonymous confirmed cases and it would complement the previous work and shed light on the fine-grained urban transmission dynamics of COVID-19. Our model is able to well fitted the whole process of fine-grained spreading pattern and the spatial-temporal spreading pattern of nine cities is stable. Our findings can be used to find the most possible infection center (spreading centroid), evaluate the growth rate, and estimate the infection risk of different communities in a new outbreak of COVID-19. Such information is very helpful for practical control and prevention. One limitation of our study is that we do not consider the impact of other social factors on city size, such as civil uprising, disobedience, and other socio-political factors. Instead, we focus on the downtown area and assume that the population is homogenous in those grids. The assumption would neglect the impact of heterogeneous socio-political factors^48–50^ and the difference in terrain height, especially in the city of Urumqi. It would be a possible intersection for future research. To have a valid conclusion here, we compare with the real trajectory data of anonymous confirmed cases and study the spreading pattern of COVID-19 in 9 cities in China, and the results turn out to be consistent. Therefore, the results have a good approximation of the fine-grained spreading process.

## Methods

### Characterize the activity centroids of confirmed cases

We adopt a collection of trajectory data contributed by anonymous smartphone users in China. The trajectory data records activity locations and corresponding timestamps when smartphone users are using location-based services. Clearly, these data reflect the real-time activity locations of smartphone users, at which they might get infected or infect others. To see how the spreading of COVID-19 is spatially correlated with the activity locations of confirmed cases, we first characterize their activity locations by a statistical metric: activity centroid (denoted by *σ*). The activity centroid of a smartphone user is defined as the average activity locations reported by this smartphone user within a given period (e.g., 1 month). That is, assuming that there are *N*_*j*_ activity locations ${P}^{j}=\{{P}_{1}^{j},{P}_{2}^{j},\cdots \,,{P}_{{N}_{j}}^{j}\}$ for a smartphone user *j*, the activity centroid *σ*_*j*_ for user *j*, is defined as ${\sigma }_{j}={\mathbb{E}}\left({P}^{j}\right)={\sum }_{k}{P}_{k}^{j}/{N}_{j}$.

**Fig. 4:**
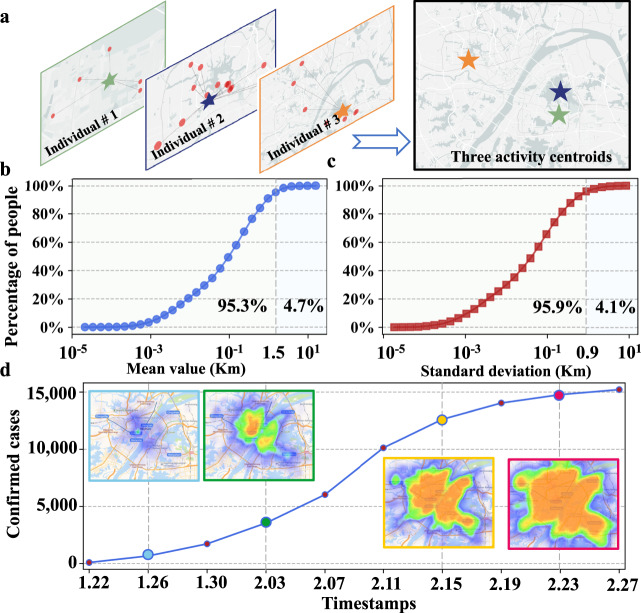
Activity centroids and visualization of COVID-19 spreading in Wuhan. **a** An illustration of calculating the activity centroids of smartphone users based on their activity locations. **b**–**c** The cumulative distributions of the mean and variance of the distance between activity centroids of each smartphone user in 1–6 months and the average centroid. **d** Visualizing the spatial-temporal spreading of COVID-19 in Wuhan between 22 January 2020 and 27 February 2020.

We illustrate the calculation of activity centroid *σ* in Fig. 4a. The activity centroid should be stable over time for most smartphone users so that it can represent the intrinsic characteristic of activity locations. For this purpose, we randomly select 20,000 smartphone users in Wuhan and calculate their activity centroids in different periods (1–6 months), obtaining 6 activity centroids *σ*_*j*_(*t*), *t* ∈ [1, 6] for each smartphone user *j*. The distances between 6 activity centroids and the average centroid (i.e., $\overline{{\sigma }_{j}}=\mathop{\sum }\nolimits_{t = 1}^{6}{\sigma }_{j}(t)/6$) for each *j* are then calculated. The mean and standard variance of these 6 distances for each user are computed and the cumulative distributions of all smartphone users are displayed in Fig. 4b, c. Clearly, the mean values of 95.3% smartphone users are <1.5 kilometer (*Km*) and the standard variance is relatively small. This implies that the activity locations of smartphone users exhibit strong stability, independent of the chosen period (see sensitivity analysis of the periods in Supplementary Fig. 5). Such an intrinsic behavior can be well captured by the activity centroid.

The most frequently visiting location (MVL) is also a statistical metric of great interest. It indicates the activity location that a smartphone user visits most frequently. We calculate the percentage of top *k* (*k* = 1, 2, ⋯ , 5) activity locations of each smartphone user and show the average of all 20,000 smartphone users in Supplementary Fig. 2a–c. The MVL (i.e., top 1 activity location) only accounts for about 45% of all activity locations, that is, more than one-half of activity location information is not utilized by MVL to characterize the activity behavior. Further, the performance of the metric of top *k* activity locations approaches that of activity centroid when *k* increases (see detailed information in Supplementary Fig. 2d–i). Therefore, we choose activity centroid as the statistical metric instead of MVL in this article.

### Definition of spreading centroids and spreading radius

To characterize the temporal spreading pattern, we divide the spreading duration in each city into *L* equal periods, and allocate confirmed cases in set *U* into subset *U*_*i*_ if their confirmation dates are within *i*th period, *i* ≤ *L*. An illustration of division is provided in the *x* axis index of Fig. 1a, c, e, where *L* = 10. Given a set *U* of all confirmed cases, we are able to calculate the overall spreading centroid (denoted by *ρ*) of *U* as the average of their activity centroids, i.e., $\rho ={\mathbb{E}}(\sigma )$, where $\sigma ={\{{\sigma }_{j}\}}_{j\in U}$. The cumulative spreading centroid is then defined as corresponding cumulative value until *i*th period, that is, the averages of activity centroids of confirmed cases in set $\mathop{\bigcup }\nolimits_{k = 1}^{i}{U}_{k}$.

We now study the spreading radius *γ* of set *U*, defined as *γ* = ∑_*j*∈*U*_*d*(*σ*_*j*_, *ρ*)/∣*U*∣, where *d*(⋅) is the Euclidean distance between the activity centroid of a confirmed case and the spreading centroid *ρ* of a set *U* of confirmed cases, and ∣*U*∣ denotes the number of confirmed cases in *U*. Obviously, the spreading radius quantifies the mean distance between confirmed cases’ activity centroids and the spreading centroid. Similarly, we introduce a cumulative spreading radius for set $\mathop{\bigcup }\nolimits_{k = 1}^{i}{U}_{i}$. The cumulative spreading radius in different periods increases slowly over time in these three cities except for periods with few cases (<10 cases).

By collaborating with Westlake Institute for Data Intelligence and local institutions for disease control and prevention, we obtain a dataset of confirmed cases who are also smartphone users for location-based services, including their activity centroids and dates of confirmation (see details on the data described in supplementary materials). We visualize the spreading process of COVID-19 in Wuhan through a heat map in Fig. 4d. The introduction of activity centroid enables us to quantify the spatial and temporal spreading pattern of COVID-19 in the following sections.

### Reporting summary

Further information on research design is available in the Nature Research Reporting Summary linked to this article.

## Supplementary information

SUPPLEMENTAL MATERIAL

Reporting Summary

## Data Availability

Activity centroids of confirmed cases in 9 cities in China we analyzed in this article and other key statistical information used in the analysis are available from data repository.

## Code availability

The code are available from code repository.
