# Supplementary material for: A general urban spreading pattern of COVID-19 and its underlying mechanism
Source: NPJ Urban Sustain. 2023 Jan 28;3(1):3. doi: 10.1038/s42949-023-00082-4 (PMC9883831; doi:10.1038/s42949-023-00082-4)
Supplement: Supplementary file 1 — SUPPLEMENTAL MATERIAL [file 42949_2023_82_MOESM1_ESM.pdf]

Supplementary Information Appendix for

A General Urban Spreading Pattern of COVID-19 and Its Underlying Mechanism

Hongshen Zhang, Yongtao Zhang, Shibo He, Yi Fang, Yanggang Cheng, Zhiguo Shi, Cunqi Shao, Chao Li, Songmin Ying, Zhenyu Gong, Yu Liu, Lin Dong, Youxian Sun, Jianmin Jia, H.Eugene Stanley, Jiming Chen

This PDF file includes:

Supplementary Notes
Supplementary Methods
Figs. S1 to S9
References for SI reference citations

Supplementary Notes

Individual trajectory data. The anonymous trajectory data used in this article was provided by Westlake Institute for Data Intelligence. Specifically, these data was collected by crowdsourcing platforms of location based services (LBS) providers. As we know, smartphone users uploaded their geographic locations with corresponding timestamps when they were having LBS (*1–10*). Such data collection was authorized by the smartphone users. To protect their privacy, their account information was hashed before they was stored on the data servers (*11*). Also, the geographic locations were stored in the form of geohash values with 8 characters. This means that geographic locations are projected into cells (rectangular cells of $38m \times 19m$, about $722m^2$) and activity locations of individuals are therefore represented by latitude and longitude of the center of corresponding cells. The data collection process complies to the Personal Information Security Specification (2017) (*12*). Note that the uploading frequency varies significantly among smartphone users. Some smartphone users upload hundreds of activity locations within a very short time, giving rise to sampling imbalance. In this article, reports of the same geographical activity locations within one hour are only counted once in order to address the sampling imbalance issue.

Selection of 9 cities. The selection of cities is mainly based on the number of confirmed cases in the cities before 2021.12. Since the effective control measures, most cities in China have less than 100 confirmed cases all over the spreading period which is nearly impossible to analyze the distribution. As shown in the Supplementary Figure 1, we need to investigate the distribution of confirmed cases within one period of infection, and we limit our condition that the city should have at least one confirmed case each day within one month. And in China mainland, there are only a total of nine cities that satisfy the condition before 2021.12.

COVID-19 confirmed cases data. All results in this article are based on the trajectory data contributed by 17,808 anonymous confirmed case in 9 cities in China. Since the outbreak of COVID-19 in China, local institutions for disease control and prevention (CDC) have been granted the authority to collect and analyze COVID-19 related data according to the Law of the People's Republic of China on Prevention and Treatment of Infectious Diseases (*13*). To facilitate our research as well as protect the privacy of COVID-19 confirmed cases, Westlake Institute for Data Intelligence first calculated the activity centroid of smartphone users and uploaded their activity centroid data and their hashed account information to the secure data servers owned by local CDC. Note that, in China, most smartphone users use their phone number as accounts. Therefore, the hashed account information means hashed phone number. Then, the phone number of COVID-19 confirmed cases on the secure data servers are also mapped into a hash value using the same hash function. Since the same phone numbers would map to the same hash values, we can use the hash values of these phone numbers to link COVID-19 confirmed cases in the trajectory data. Before the data is output and sent to us for further analysis, all the account information are anonymized again by random IDs to prevent ID tracking. Therefore the final output data for analysis contains the activity centroid and date of confirmation of each anonymous COVID-19 confirmed case with a random ID. We emphasize that all individual-

level information regarding COVID-19 confirmed cases are computed and analyzed by local CDC on their secure data servers. We do not have any detailed information about COVID-19 confirmed cases. The whole procedure, including data collection, storage and analysis, is subject to laws of both China and America (*13–15*). Moreover, this project was approved by the Ethics Committee of school of Medicine, Zhejiang University.

Flowchart of research. As shown in Supplementary Figure 2, the flowchart of our research includes four steps. First, confirmed cases' trajectory data were collected by crowdsourcing platforms of location based services(LBS) providers. Second, Westlake Institute for Data Intelligence calculated the activity centroids of confirmed cases. Third, the spatial and temporal spreading pattern was analyzed based on spreading centroid and spreading radius. Finally, combining the impact of mobility pattern, policy measure, population size, and city size, we reconstructed the fine-grained urban spreading process and provided policy recommendations.

Supplementary Methods

Evaluation for activity centroid The activity centroid is introduced in this article to represent the statistics of activity locations of each COVID-19 patient. Compared with the most frequently visiting activity location, activity centroid is more stable over time. To demonstrate the advantages of activity centroid, we first calculate the percentages of top 1 to top 5 activity locations of 20,000 smartphone users in each city respectively, as shown in Supplementary Figures 3a-3c. Note that, the set of top k activity locations of each user is the sum of all top i ($i = 1, 2, \cdots, k$) activity locations. The figure shows that in Wuhan, Beijing and Urumqi, the most frequently visiting activity location (top 1) only accounts for 45% of all activity locations of smartphone users, which inevitably leads to the underutilization of massive location-related information. We attempt to address the issue by incorporating more activity location data, but unfortunately, the top 5 activity locations accounts for about 80% of all activity locations. To show the connection between activity centroid and the top k ($k = 1, 2, \cdots, 5$) activity locations, we calculate the average of difference between the activity centroid and the top k activity locations (see Supplementary Figure 3d-3f). It is shown that as k increases, the difference decreases. This indicates that the performance of top k activity locations approaches that of activity centroid as k increases. Therefore, activity cetroid has a better performance in representing the activity positions of smartphone users than the metric of top k activity location. We also plot the spreading radius based on activity centroid and the top k activity locations in Supplementary Figures 3g-3i, which yields similar conclusion.

Sensitivity analysis on temporal spreading pattern of COVID-19 In this section, we conduct a series of sensitivity analysis on key parameters used for analyzing temporal spreading pattern of COVID-19. We first examine the impact of sampling period (i.e., the duration in which activity locations were collected before confirmed cases were clinically confirmed) of activity centroid on the spreading centroid and spreading radius. The sampling period is set as 30 days, 90 days and 180 days and the corresponding activity locations of all users are collected.

Based on the data above, we recalculate the spreading centroid and spreading radius of Wuhan, Beijing and Urumqi, respectively. As shown in Supplementary Figure 4, spreading centroid and spreading radius vary slightly in different sampling periods, and almost have the same tendency. Specifically, spreading centroid of three cites in different sampling periods fluctuate in a very small scope: the differences among three periods are less than 0.2 Km in Wuhan, less than 0.5 Km in Beijing and 1.0 Km in Urumqi (Supplementary Figures 4a-4c), respectively. Since the number of confirmed cases in Beijing and Urumqi is significantly less than that in Wuhan, the tendency in Beijing and Urumqi are relatively unstable. Considering the city size of Beijing and Urumqi are larger than 8,000 Km^2, fluctuations in these two cites are reasonable. Again, the differences of spreading radius under different sampling periods in these three cities are also small (Supplementary Figures 4d-4f). Moreover, the spreading centroids in different sampling periods have strong similarities. To analyze the temporal spreading pattern, we divide the spreading duration into L equal periods. The results of $L = 10$ are provided in Fig. 2. We proceed to analyze the impact of L on the temporal spreading pattern of COVID-19. We set $L = 5$ and $L = 15$ respectively, and calculate the cumulative spreading centroid and the spreading radius of Wuhan, Beijing and Urumqi. From Supplementary Figures 5a and 5d, it can be seen that the difference of cumulative spreading radius in Wuhan at the first point under $L = 5$ and $L = 15$ is relatively large due to the limited number of confirmed cases. Most of time, cumulative spreading radius in different periods are quite similar. The same conclusion can be drawn from the results of spreading radius in Beijing and Urumqi (Supplementary Figures 5b, 5c, 5e and 5f). This shows that the selection of L does not have much impact on tendency of spreading radius. We also calculate the cumulative spreading centroids of these three cities under $L = 5$ and $L = 15$ (Supplementary Figures 5g-5l). When L becomes larger, which means each period becomes shorter, the mean abstract error (MAE) becomes larger. However, MAEs in all cases is less than 0.9 Km, showing that the spreading centroid is not sensitive to L.

Results of temporal spreading pattern in other 6 cities In order to have a comprehensive understanding of the city-level temporal spreading pattern, we perform the same analysis of temporal spreading pattern in other 6 cities in China, i.e., Xiaogan, Suizhou, Xiangyang, Huanggang, Guangzhou and Wenzhou. Similar to the analyzing process of Wuhan, Beijing and Urumqi, we divide the spreading duration in each city into 10 equal periods and then compute the differences between the cumulative spreading centroid until i^{th} period and the overall spreading centroid in Supplementary Figures 6a-6f. It indicates that the cumulative spreading centroids in Xiaogan, Suizhou, Xiangyang, Huanggang in different periods are very close to the overall spreading centroid (the MAE of the difference is less than 0.3 Km). However, we see relatively large MAEs of cumulative spreading centroid in Guangzhou and Wenzhou, mainly because these two cities are far away from Wuhan and have many imported cases at railway stations and airports that are sparsely scattered. We proceed to study the spreading radius in these 6 cities. As shown in Supplementary Figures 6g-6l, except for Xiaogan, the cumulative spreading radius increases slowly over time in most periods in all 5 cities. Since there are few confirmed cases (less than 10 cases) in the first period of Xiaogan, it may cause randomness and result in much higher cumulative radius at the first period. Similarly, the same condition

appears in Xiangyang and Huanggang, but less severely.

Results of spatial spreading pattern in other 6 cities In this section, we discuss the spatial spreading pattern of COVID-19 in detail, that is, city-level spatial distribution of activity centroid of COVID-19 confirmed cases. To characterize and visualize spatial spreading pattern intuitively, we first discretize the geographical area of each city into grids of $1Km \times 1Km$, in which the overall spreading centroid is chosen as the center for the grids. Then, we label each grid by calculating distance between grids in the area and the central grid, that is, the grid including overall spreading centroid. Confirmed cases is then projected into the grids and allocated to corresponding grid according to their activity centroids. Statistical results for Xiaogan, Suizhou, Xiangyang, Huanggang, Guangzhou and Wenzhou are illustrated in Supplementary Figures 7 and 8, in which a dot i with coordinate of (x_i, y_i) represents that an average of y_i confirmed cases are in the grid whose distance from the overall spreading centroid satisfies $x_i \leq d < (x_i + 1)$. Therefore, $y_i = N_c/N_g$, where N_g represents the number of grids satisfying $(x_i - 1) \leq d < x_i$ and N_c represents the total number of confirmed cases distributed in these grids. As we can see, the spatial distributions for Beijing, Urumqi, Xiaogan, Suizhou and Xiangyang exhibit a prominent linear pattern, indicating that the spatial distributions in these cities follow power-law distribution approximately. Different from these cities, the spatial distribution of confirmed cases in Xiangyang does not completely follow power-law distribution: points deviate from the fitted regression line when d is small. We also notice that for cities (such as Guangzhou and Wenzhou) where imported cases are widely scattered, the spatial spreading pattern is less prominent. It is clear to see that there are multiple clusters of confirmed cases in these two cities (Supplementary Figure 8), which impacts the spatial spreading. Combining the observations for spatial and temporal spreading, we conclude that one main infection source is a necessary condition for COVID-19 to have a clear power-law-like spreading pattern in a city.

The mechanism for Spatial-Temporal Spreading Pattern To account for mechanism underlying the phenomenon we observed from the data of confirmed cases in 9 cities in China, we introduce the Kendall model. By generalizing the Kermack-McKendrick model to spatially dependent integro-differential equations, spatial spreading pattern of infectious diseases could be well captured by the Kendall model, which is formulated by

$$\frac{\partial S}{\partial t} = -\beta S(x,t) \int_{-\infty}^{\infty} I(y,t) K(x-y) \mathrm{d}y \tag{1}$$

$$\frac{\partial I}{\partial t} = \beta S(x,t) \int_{-\infty}^{\infty} I(y,t) K(x-y) \mathrm{d}y - \gamma I(x,t) \tag{2}$$

$$\frac{\partial R}{\partial t} = \gamma I(x,t) \tag{3}$$

where $S(x,t)$, $I(x,t)$, $R(x,t)$ represent numbers of susceptible, infected and recovered individuals at location x and time t. β and γ represent infected and recovered rate, respectively. The kernel function $K(x-y) > 0$ weights the contributions of the confirmed cases at location y to the susceptible cases at location x, satisfying $\int_{-\infty}^{\infty} K(y)\mathrm{d}y = 1$. By rescaling the initial model,

we obtain the following normalized differential equation

$$\frac{\partial S}{\partial t} = -S(x,t)\int_{-\infty}^{\infty} I(y,t)K(x-y)\mathrm{d}y \tag{4}$$

$$\frac{\partial I}{\partial t} = S(x,t)\int_{-\infty}^{\infty} I(y,t)K(x-y)\mathrm{d}y - \lambda I(x,t) \tag{5}$$

$$\frac{\partial R}{\partial t} = \lambda I(x,t) \tag{6}$$

Combining equation (4)-(6), we write

$$\frac{\partial R}{\partial t} = -\lambda R(x,t) + \lambda\left[1 - \exp\left(-\frac{1}{\lambda}\int_{-\infty}^{\infty} R(y,t)K(x-y)\mathrm{d}y\right)\right] + \lambda I_0(x) \tag{7}$$

where $S(x,t)$, $I(x,t)$, $R(x,t)$ denote the proportion of individuals in three kinds of statuses at location x and time t. Hence, $S(x,t) + I(x,t) + R(x,t) = 1$. Besides, $\lambda = \gamma/\beta\xi$, where ξ represents the number of initial susceptible individuals in each grid and $\xi \times R(x,t)$ therefore represents the number of recovered individuals calculated from simulation. Note that kernel function $K(x-y)$ weights the contributions of the infected individuals at location y (x) to the infection of the susceptible individuals at location x (y). According to previous study (*16*), K can be expressed by a power-law distribution:

$$K(\Delta r) = \Delta r^{\eta} \tag{8}$$

where Δr represents the traveling distance, η denote power-law exponential, respectively. Based on equation (8), we calculate the probability of infected individuals traveling between any two locations, and therefore further quantify the impact of human mobility pattern on COVID-19 spatial-temporal spreading pattern. We uniformly define the $0.1Km$ as the minimum measurement subsection and view the travel distance less than $0.1Km$ as $0.1Km$ to avoid singularity point at $\Delta r = 0$. To determine the power-law exponential parameter η in kernel function, we select 20,000 individuals for Wuhan, Beijing and Urumqi, respectively, and count the number of travels with different distances. Statistical results for three cities are illustrated in Figs. 3d-3f and we could see obviously change in human mobility pattern after control measures in Wuhan (Jan .23) and Urumqi (Jul .16). Here we haven't utilize the well-known truncated power-law distribution (*16*) to describe the human mobility pattern since that the city range we analysed is less than $50Km$ which is much less than the truncated distance level (larger than $200Km$).

Note that COVID-19 confirmed cases in China get isolated for medical treatment and cannot cause further infection once they are confirmed. Therefore, confirmed cases can be regarded as recovered individuals by $\xi \times R(x,t)$. We therefore fit equation (7) based on information of confirmed cases we collected, including the date of confirmation and activity centroids of data of confirmed cases. In order to simulate the spreading of COVID-19 in discrete geographic

area (*17*), we first set spreading centroid calculated in advance as the spreading origin (i.e., (0,0)) for each city, and then divide whole city into grids of size $1Km\times 1Km$. Since the longest distance between activity centroids of confirmed cases and spreading centroid in Wuhan, Beijing and Urumqi are 50 Km, 35 Km, 30 Km. Thus, we discretize areas of these three cites with 101 $\times$ 101(ranging from [-50,50] $\times$ [-50,50]), 71 $\times$ 71 (ranging from [-35,35] $\times$ [-35,35]) and 61 $\times$ 61(ranging from [-30,30] $\times$ [-30,30]) grids, respectively. It is also worth noticing that we assume all initial infected individuals originates from center of the area $(0,0)$. Altogether, some parameters are determined using trajectory data of smartphone users, including η in kernel function, incubation period (median 5 days (*18, 19*)), recovery rate (γ=1/2.9 (*20–25*)) and the number of susceptible individuals in each grid ($\psi = N/N_g$, N_g represents the number of divided grids and N represents total population in each city, N for Wuhan, Beijing, Urumqi are 10,000,000, 20,000,000 and 3,800,000 respectively (*20*)). Since there are lock down period for Wuhan at Jan .23 and for Urumqi at Jul .16 where resulted in different human mobility pattern, simulation of Wuhan and Urumqi have two different stages with different η. The spreading duration which contains most of confirmed cases in Wuhan, Beijing and Urumqi are 40 days, 30 days and 20 days. With these inputs, we obtain a set of optimal parameters (λ and I_0) by using the lsqcurvefit function of the Matlab optimization toolbox (*26*) to fit real data of each city. Combining the fitted parameter λ, we obtain basic reproduction number R_0 for Wuhan before the lock down period and after the lock down period is 2.02 and 1.09, for Beijing in whole period is 0.23 and for Urumqi before the lock down and after the lock down period is 1.35 and 1.06. As shown in Supplementary Figure 9a - Fig. 9f and Fig. 4a - Fig. 4c, Kendall model well captures the evolution of recovered individuals in Wuhan , Beijing and Urumqi from both spatial and temporal perspective. Altogether, these simulation results both demonstrates the good performance of our model to capture the special spatial-temporal urban spreading pattern of COVID-19.

References

1. Thurner, S., Klimek, P. & Hanel, R. A network-based explanation of why most covid-19 infection curves are linear. *Proc. Natl. Acad. Sci. U.S.A.* **117**, 22684–22689 (2020).

2. Persson, J., Parie, J. F. & Feuerriegel, S. Monitoring the covid-19 epidemic with nationwide telecommunication data. *Proc. Natl. Acad. Sci. U.S.A.* **118** (2021).

3. Han, X. *et al.* Quantifying covid-19 importation risk in a dynamic network of domestic cities and international countries. *Proc. Natl. Acad. Sci. U.S.A.* **118** (2021).

4. Vigfusson, Y. *et al.* Cell-phone traces reveal infection-associated behavioral change. *Proc. Natl. Acad. Sci. U.S.A.* **118** (2021).

5. Xiong, C., Hu, S., Yang, M., Luo, W. & Zhang, L. Mobile device data reveal the dynamics in a positive relationship between human mobility and covid-19 infections. *Proc. Natl. Acad. Sci. U.S.A.* **117**, 27087–27089 (2020).

6. Kido, H., Yanagisawa, Y. & Satoh, T. An anonymous communication technique using dummies for location-based services. In *ICPS'05. Proceedings. International Conference on Pervasive Services, 2005.*, 88–97 (IEEE, 2005).

7. Küpper, A. *Location-based services: fundamentals and operation* (John Wiley & Sons, 2005).

8. Phillips, A. *et al.* Location-based services (2010). US Patent 7,848,765.

9. Schiller, J. & Voisard, A. *Location-based services* (Elsevier, 2004).

10. Gruteser, M. & Grunwald, D. Anonymous usage of location-based services through spatial and temporal cloaking. In *Proceedings of the 1st international conference on Mobile systems, applications and services*, 31–42 (2003).

11. Mihcak, M. K. & Venkatesan, R. Hash value computer of content of digital signals (2007). US Patent 7,240,210.

12. Wang Han, S. & Munir, A. B. Information security technology-personal information security specification: China's version of the gdpr. *Eur. Data Prot. L. Rev.* **4**, 535 (2018).

13. Liang, H. *The Draft Civil Code of the People's Republic of China: English Translation (Prepared by the Legislative Research Group of the Chinese Academy of Social Sciences)* (Brill, 2010).

14. Voigt, P. & Von dem Bussche, A. The eu general data protection regulation (gdpr). *A Practical Guide, 1st Ed., Cham: Springer International Publishing* (2017).

15. Jones, W. C. A translation of the fourth draft civil code (june 1982) of the people's republic of china. *Rev. Social. Law* **10**, 193–193 (1984).

16. Gonzalez, M. C., Hidalgo, C. A. & Barabasi, A.-L. Understanding individual human mobility patterns. *Nature* **453**, 779–782 (2008).

17. Schneckenreither, G., Popper, N., Zauner, G. & Breitenecker, F. Modelling sir-type epidemics by odes, pdes, difference equations and cellular automata–a comparative study. *Simul. Model. Pract. Theory* **16**, 1014–1023 (2008).

18. Lauer, S. A. *et al.* The incubation period of coronavirus disease 2019 (covid-19) from publicly reported confirmed cases: estimation and application. *Ann. Intern. Med.* **172**, 577–582 (2020).

19. Linton, N. M. *et al.* Incubation period and other epidemiological characteristics of 2019 novel coronavirus infections with right truncation: a statistical analysis of publicly available case data. *J. Clin. Med.* **9**, 538 (2020).

20. Hao, X. *et al.* Reconstruction of the full transmission dynamics of covid-19 in wuhan. *Nature* **584**, 420–424 (2020).

21. Wu, J. T. *et al.* Estimating clinical severity of covid-19 from the transmission dynamics in wuhan, china. *Nat. Med.* **26**, 506–510 (2020).

22. Wu, J. T., Leung, K. & Leung, G. M. Nowcasting and forecasting the potential domestic and international spread of the 2019-ncov outbreak originating in wuhan, china: a modelling study. *Lancet* **395**, 689–697 (2020).

23. He, X. *et al.* Temporal dynamics in viral shedding and transmissibility of covid-19. *Nat. Med.* **26**, 672–675 (2020).

24. Li, Q. *et al.* Early transmission dynamics in wuhan, china, of novel coronavirus–infected pneumonia. *N. Engl. J. Med.* (2020).

25. Bi, Q. *et al.* Epidemiology and transmission of covid-19 in 391 cases and 1286 of their close contacts in shenzhen, china: a retrospective cohort study. *Lancet Infect. Dis.* (2020).

26. Hallauer, W., Slemp, W. & Kapania, R. Curve fitting free-vibration time response for estimation of structural dynamic parameters. *Exp. Tech.* **34**, 26–33 (2010).

Table R1 | Number of accumulated confirmed cases within a city

COVID-19 spreading in China

City Name	Confirmed cases	Main Spreading Period
Wuhan	50423	2020.1 – 2020.4
Beijing	293	2020.6 – 2020.8
Urumqi	856	2020.7 – 2020.9
Xiaogan	3518	2020.1 – 2020.4
Suizhou	1307	2020.1 – 2020.4
Xiangyang	1175	2020.1 – 2020.4
Huanggang	2913	2020.1 – 2020.4
Guangzhou	887	2020.2 – 2020.5
Wenzhou	516	2020.2 – 2020.6
**Other well-known cities**		
Shanghai	59068	2022.3 – 2022.5
Jilin	40242	2022.1 – 2022.5

Supplementary Figure 1. Number of Confirmed cases in China.

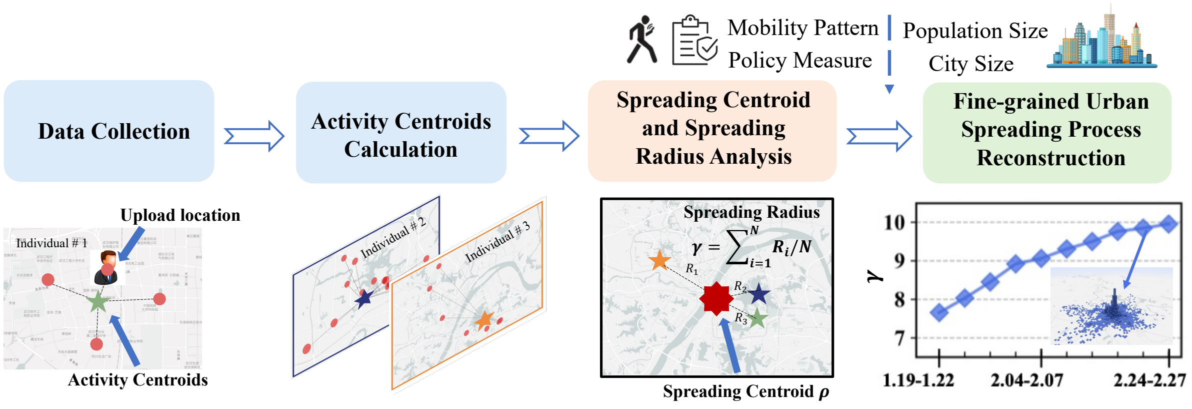

Supplementary Figure 2. Flowchart including data collection, data calculation, data analysis and spreading process reconstruction.

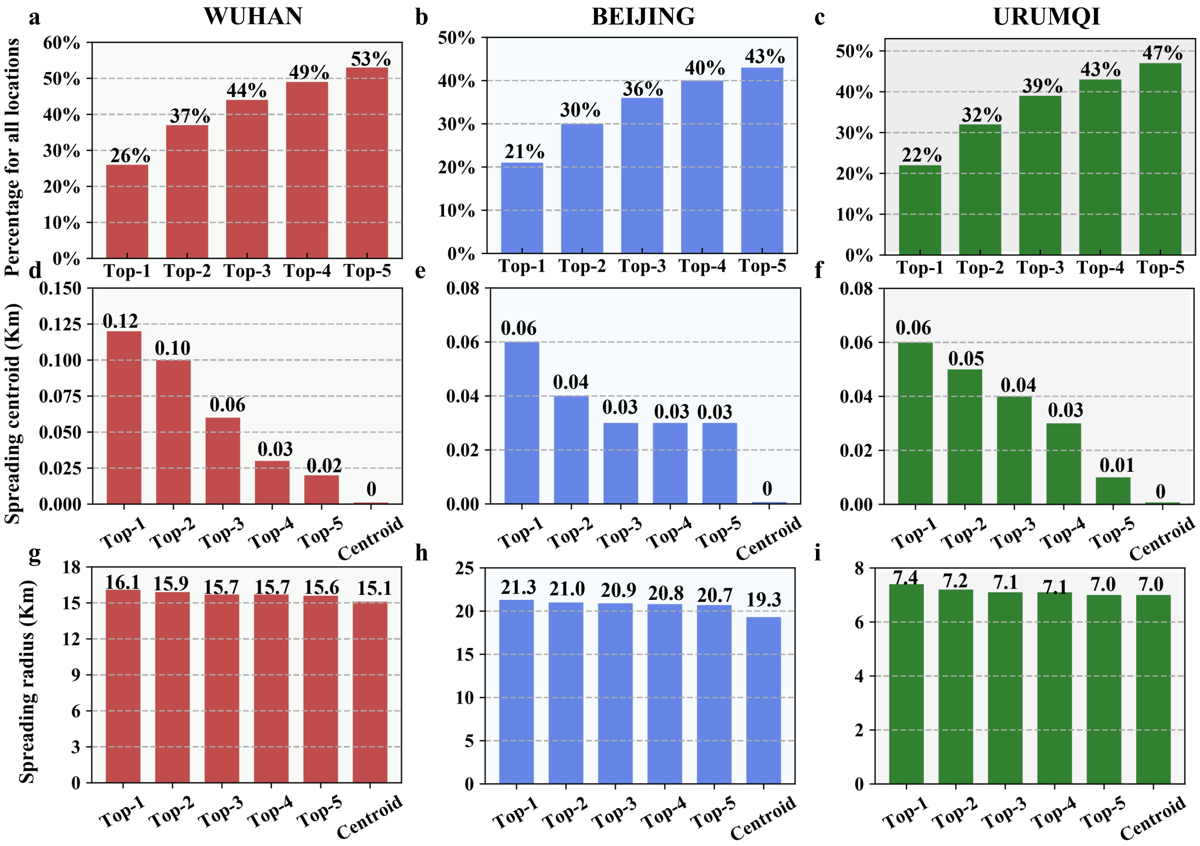

Supplementary Figure 3. Detailed evaluation for activity centroid. a to **c** Percentage of top 1 to top 5 account for all activity locations that people in Wuhan, Beijing and Urumqi have been uploaded. **d** to **f** Distance between spreading centroids calculated based on activity centroid and spreading centroids calculated based on top 1 to top 5 activity locations in Wuhan, Beijing and Urumqi. **g** to **i** Spreading radius of Wuhan, Beijing and Urumqi calculated based on activity centroid and top 1 to top 5 activity locations respectively.

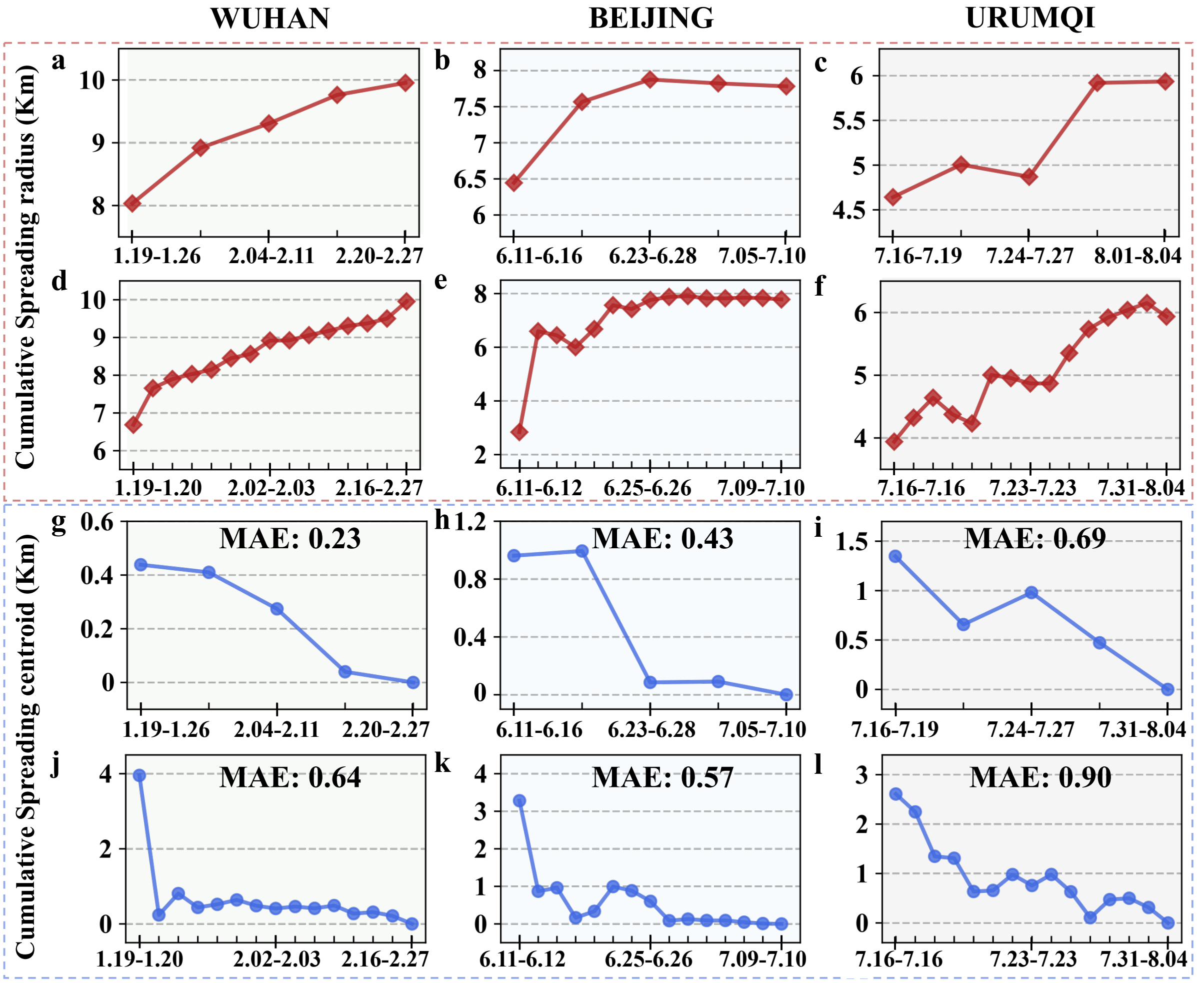

Supplementary Figure 4. Sensitivity analysis on sampling period. a to **c** Cumulative spreading centroids for Wuhan, Beijing and Urumqi under different sampling periods. **d** to **f** Cumulative spreading radius for Wuhan, Beijing and Urumqi under different sampling periods.

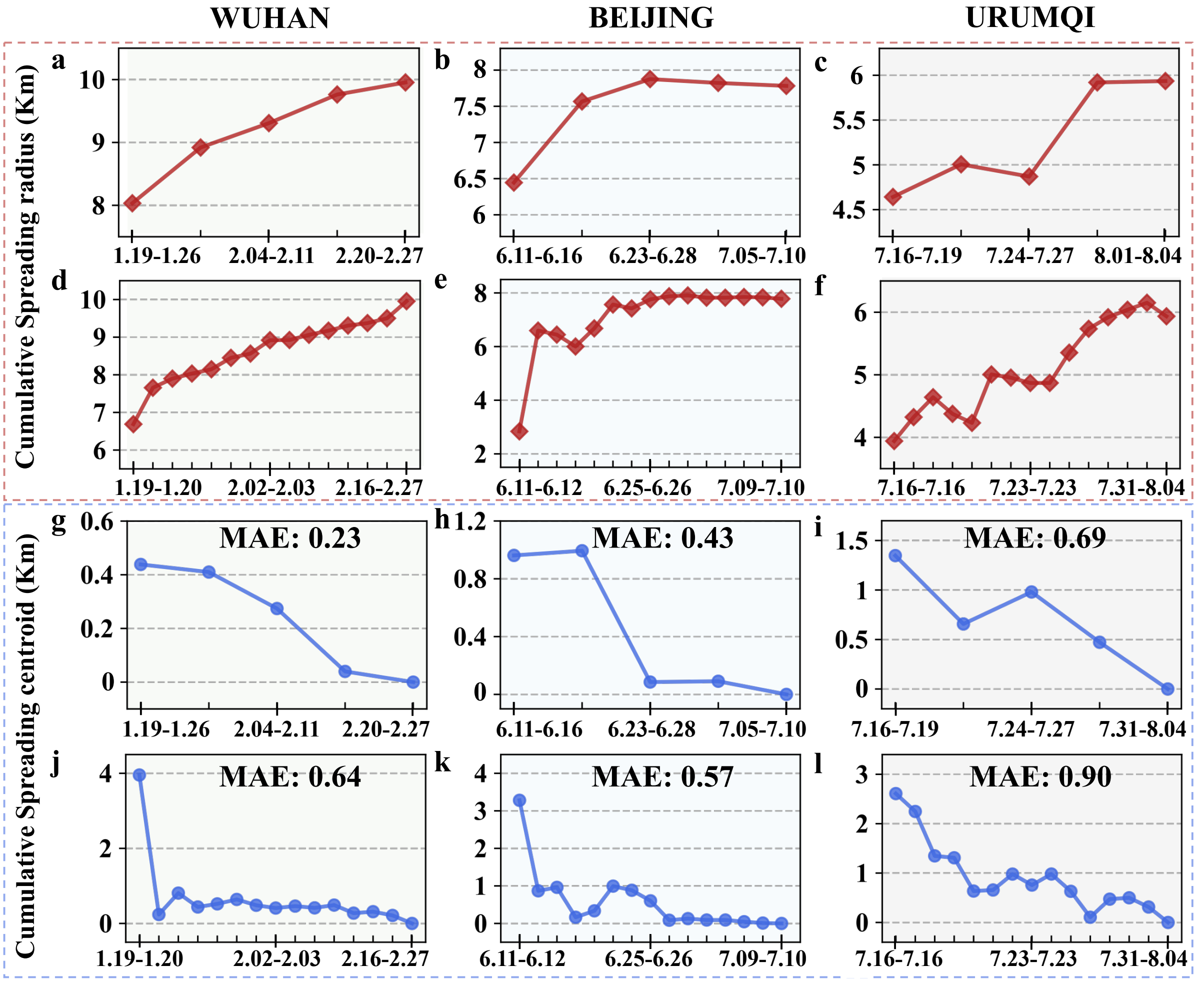

Supplementary Figure 5. Sensitivity analysis on the number of spreading duration period (L). a to **f** Cumulative spreading radius for Wuhan, Beijing and Urumqi under $L = 5$ and $L = 15$, respectively. **g** to **l** Cumulative spreading centroids for Wuhan, Beijing and Urumqi under $L = 5$ and $L = 15$, respectively.

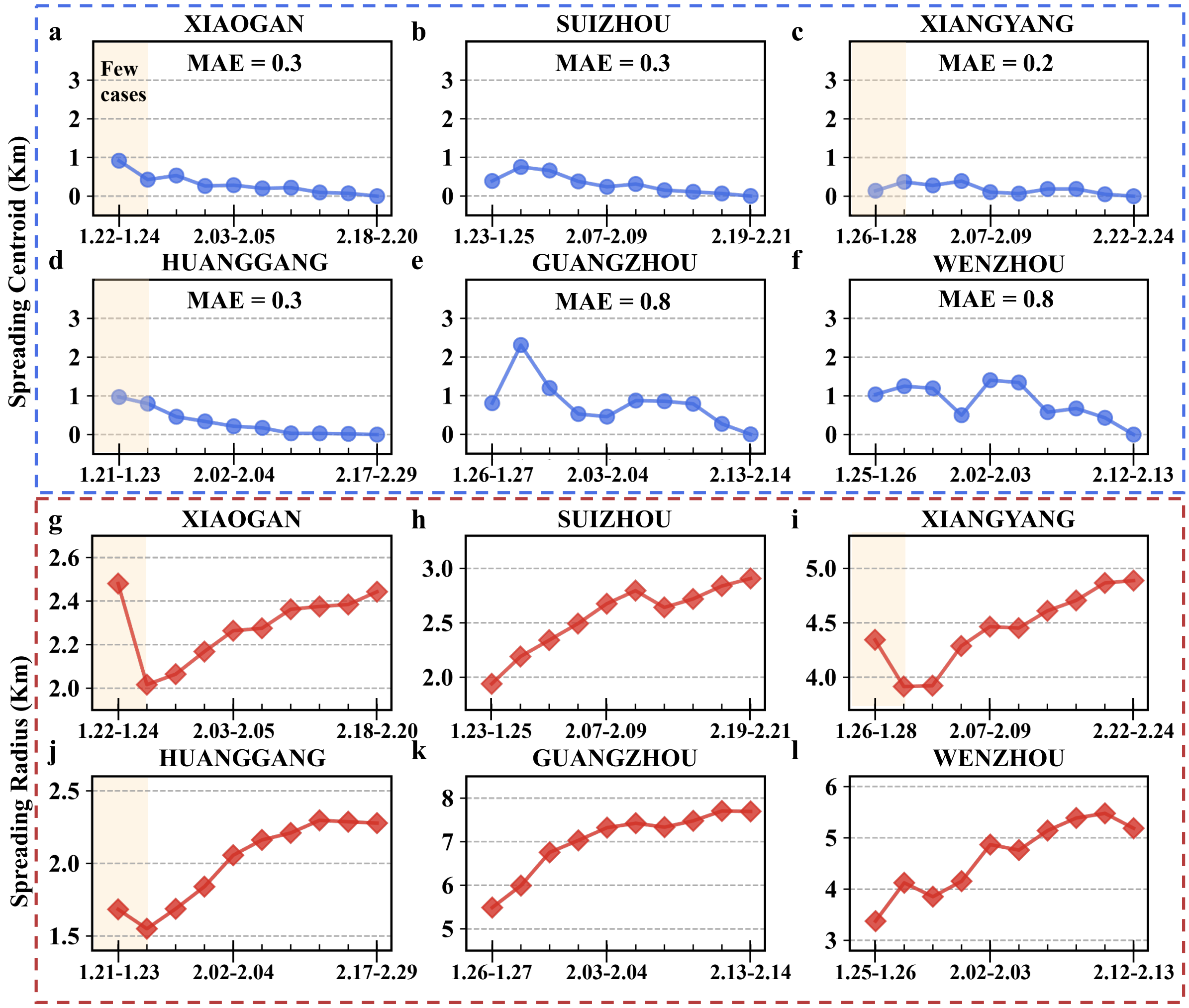

Supplementary Figure 6. The temporal spreading pattern of COVID-19 in Xiaogan, Suizhou, Xiangyang, Huanggang, Guangzhou and Wenzhou. a to **f** The cumulative spreading centroids for Xiaogan, Suizhou, Xiangyang, Huanggang, Guangzhou and Wenzhou, respectively. **g** to **l** The cumulative spreading radius in these six cities.

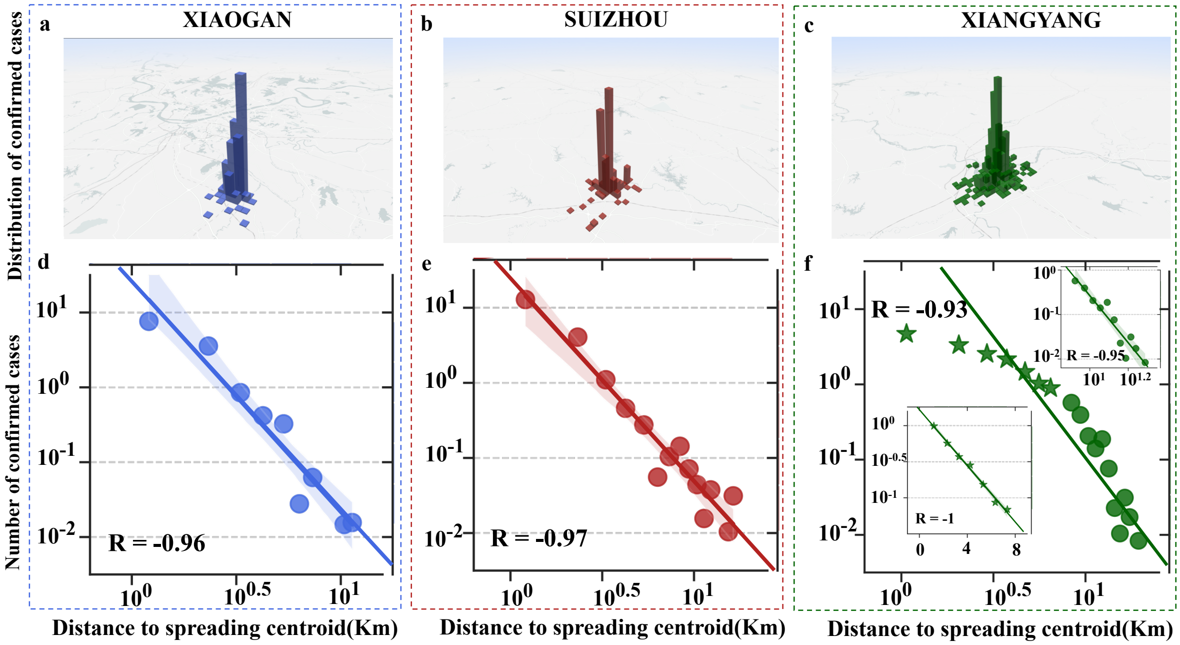

Supplementary Figure 7. The spatial spreading pattern of COVID-19 in Xiaogan, Suizhou and Xiangyang. a to **c** A visualization of the number of confirmed cases in discretized grids in Xiaogan, Suizhou and Xiangyang, respectively. **d** to **f** The spatial distributions (dots) as a function of distance from spreading centroid and the fitted regression lines for these distributions.

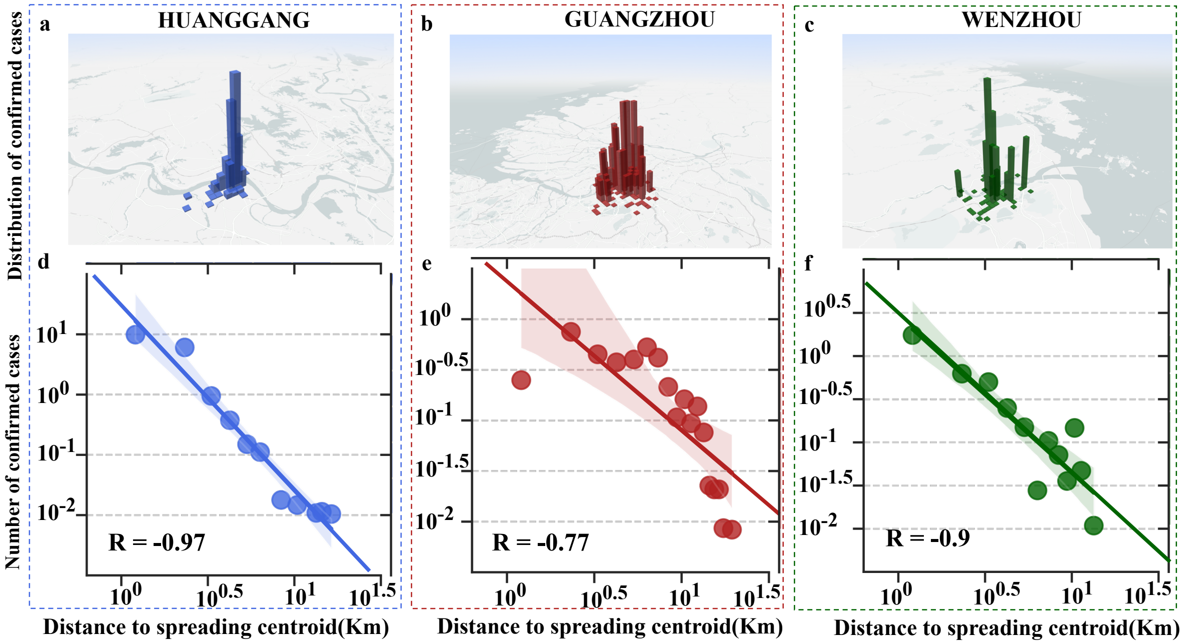

Supplementary Figure 8. The spatial spreading pattern of COVID-19 in Huanggang, Guangzhou and Wenzhou, respectively. **a** to **c** A visualization of the number of confirmed cases in discretized grids in Huanggang, Guangzhou and Wenzhou, respectively. **d** to **f** The spatial distributions (dots) as a function of distance from overall spreading centroid and the fitted regression lines for these distributions.

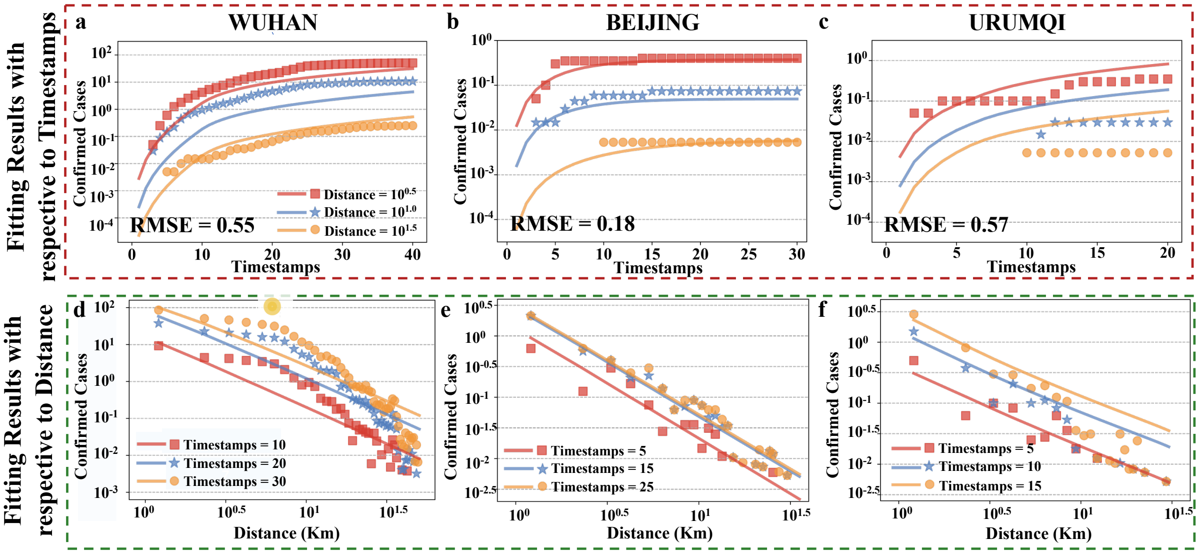

Supplementary Figure 9. Analysis on three key parameters in Kendall model. a to **c** The fitting performance with respective to temporal dimension. **d** to **f** The fitting performance with respective to spatial dimension.
