# Supplementary material for: A general urban spreading pattern of COVID-19 and its underlying mechanism
Source: NPJ Urban Sustain. 2023 Jan 28;3(1):3. doi: 10.1038/s42949-023-00082-4 (PMC9883831; doi:10.1038/s42949-023-00082-4)
Supplement: Supplementary file 2 — Reporting Summary [file 42949_2023_82_MOESM2_ESM.pdf]

nature portfolio

Corresponding author(s): Shibo He, Jiming Chen

Last updated by author(s): Dec 7, 2022

Reporting Summary

Nature Portfolio wishes to improve the reproducibility of the work that we publish. This form provides structure for consistency and transparency in reporting. For further information on Nature Portfolio policies, see our Editorial Policies and the Editorial Policy Checklist.

Statistics

For all statistical analyses, confirm that the following items are present in the figure legend, table legend, main text, or Methods section.

n/a	Confirmed	
☐	☒	The exact sample size (n) for each experimental group/condition, given as a discrete number and unit of measurement
☒	☐	A statement on whether measurements were taken from distinct samples or whether the same sample was measured repeatedly
☒	☐	The statistical test(s) used AND whether they are one- or two-sided<br>*Only common tests should be described solely by name; describe more complex techniques in the Methods section.*
☒	☐	A description of all covariates tested
☒	☐	A description of any assumptions or corrections, such as tests of normality and adjustment for multiple comparisons
☒	☐	A full description of the statistical parameters including central tendency (e.g. means) or other basic estimates (e.g. regression coefficient) AND variation (e.g. standard deviation) or associated estimates of uncertainty (e.g. confidence intervals)
☒	☐	For null hypothesis testing, the test statistic (e.g. F, t, r) with confidence intervals, effect sizes, degrees of freedom and P value noted<br>*Give P values as exact values whenever suitable.*
☒	☐	For Bayesian analysis, information on the choice of priors and Markov chain Monte Carlo settings
☒	☐	For hierarchical and complex designs, identification of the appropriate level for tests and full reporting of outcomes
☐	☒	Estimates of effect sizes (e.g. Cohen's d, Pearson's r), indicating how they were calculated

Our web collection on statistics for biologists contains articles on many of the points above.

Software and code

Policy information about availability of computer code

Data collection	We obtained via our industry partners the anonymous activity centroids, which is defined as the average of activity locations reported by infectious individuals. The activity centroids used in the analysis have been made available for review.
Data analysis	All codes used in the analysis are programmed originally; Software includes Python (V.3.7.7, Python Software Foundation) and MATLAB(version 2020a). We have made the codes available for review.

For manuscripts utilizing custom algorithms or software that are central to the research but not yet described in published literature, software must be made available to editors and reviewers. We strongly encourage code deposition in a community repository (e.g. GitHub). See the Nature Portfolio guidelines for submitting code & software for further information.

Data

Policy information about availability of data

All manuscripts must include a data availability statement. This statement should provide the following information, where applicable:

- Accession codes, unique identifiers, or web links for publicly available datasets
- A description of any restrictions on data availability
- For clinical datasets or third party data, please ensure that the statement adheres to our policy

The anonymized activity centroids and all codes used in all analyses have been provided.

Human research participants

Policy information about studies involving human research participants and Sex and Gender in Research.

Reporting on sex and gender	We used the anonymized activity centroids extracted from crowdsourced data from 1 January, 2020 to 4 August, 2020. The anonymized activity centroids were extracted from crowdsourced data contributed by voluntary users, covering approximately 2/3 of confirmed cases in Wuhan, Beijing, Urumqi, Xiaogan, Suizhou, Xiangyang, Huanggang, Gaungzhou, Wenzhou.
Population characteristics	The confirmed cases from 1 January, 2020 to 4 August, 2020 in Wuhan, Beijing, Urumqi, Xiaogan, Suizhou, Xiangyang, Huanggang, Guangzhou and Wenzhou.
Recruitment	(Not applicable) The activity centroids were provided by our industry partners.
Ethics oversight	We do not use any identified individual-level data, but anonymized activity centroids. This work was approved of ethics review from Medical Ethics Committee of School of Medicine, Zhejiang University.

Note that full information on the approval of the study protocol must also be provided in the manuscript.

Field-specific reporting

Please select the one below that is the best fit for your research. If you are not sure, read the appropriate sections before making your selection.

☐ Life sciences ☒ Behavioural & social sciences ☐ Ecological, evolutionary & environmental sciences

For a reference copy of the document with all sections, see nature.com/documents/nr-reporting-summary-flat.pdf

Behavioural & social sciences study design

All studies must disclose on these points even when the disclosure is negative.

Study description	We explore the anonymized activity centroids to reveal the general spreading pattern of COVID-19.
Research sample	We used the activity centroids extracted from crowdsourced data from 1 January, 2020 to 4 August, 2020 in Wuhan, Beijing, Urumqi, Xiaogan, Suizhou, Xiangyang, Huanggang, Guangzhou and Wenzhou.
Sampling strategy	The activity centroids were extracted from crowdsourced data contributed by voluntary users, covering approximately 2/3 of confirmed cases in Wuhan, Beijing, Urumqi, Xiaogan, Suizhou, Xiangyang, Huanggang, Guangzhou and Wenzhou.
Data collection	We obtained via our industry partners the anonymized activity centroid, which is defined as the average of activity locations reported by confirmed cases. The activity centroids used in the analysis have been made available for review.
Timing	The trajectory data was collected during the period 1 December, 2019 to 4 July, 2020; The confirmed case data was collected starting from 1 January, 2020 to 4 August, 2020.
Data exclusions	Activity centroids whose user had uploaded activity location within one month before confirmation have been included for analysis, while users who did not have activity location within one month are excluded.
Non-participation	(Not applicable) We used the activity centroids extracted from crowdsourced data at Westlake Institute for Data Intelligence during the study period.
Randomization	(Not applicable) There is no experimental condition since this study was not an experiment.

Reporting for specific materials, systems and methods

We require information from authors about some types of materials, experimental systems and methods used in many studies. Here, indicate whether each material, system or method listed is relevant to your study. If you are not sure if a list item applies to your research, read the appropriate section before selecting a response.

Materials & experimental systems

n/a	Involved in the study
☒	☐ Antibodies
☒	☐ Eukaryotic cell lines
☒	☐ Palaeontology and archaeology
☒	☐ Animals and other organisms
☒	☐ Clinical data
☒	☐ Dual use research of concern

Methods

n/a	Involved in the study
☒	☐ ChIP-seq
☒	☐ Flow cytometry
☒	☐ MRI-based neuroimaging
